# Exploring the Evolving Role of Pharmaceutical Services in Community Pharmacies: Insights from the USA, England, and Portugal

**DOI:** 10.3390/healthcare13151786

**Published:** 2025-07-23

**Authors:** M. Luísa G. Cunha Leal, Ana Rita Rodrigues, Victoria Bell, Mário Forrester

**Affiliations:** 1 Social Pharmacy and Public Health Laboratory, Faculty of Pharmacy, University of Coimbra, 3000-548 Coimbra, Portugal; luisacunhaleal@gmail.com (M.L.G.C.L.); artrodrigues@ff.uc.pt (A.R.R.); 2 Associated Laboratory for Green Chemistry of the Network of Chemistry and Technology (LAQV-REQUIMTE), Group of Pharmaceutical Technology, Faculty of Pharmacy, University of Coimbra, 3000-548 Coimbra, Portugal; 3 UFUP—Pharmacovigilance Unit of the University of Porto, 4200-450 Porto, Portugal

**Keywords:** community pharmacy, pharmaceutical services, pharmaceutical care, healthcare systems, pharmacists

## Abstract

Chronic diseases are a leading cause of death worldwide and have a significant negative impact on public health, overall well-being, national economies, and the long-term sustainability of already burdened health systems. In addressing some of the current health challenges, the contribution of pharmacists and community pharmacies is of particular significance. Pharmacists play a vital role in the medication use process, enhancing the efficacy of pharmacological interventions and facilitating the delivery of health services. Community pharmacies occupy a key position within the healthcare system, acting as a primary point of contact with the public and frequently representing the most accessible healthcare facility for patients. In recent times, community pharmacies have undergone a process of adaptation, shifting from a narrow focus on the dispensing of medications towards a more comprehensive approach that is patient-centered and incorporates a range of healthcare services, while also prioritizing the quality of the services provided. This work aims to explore the role of pharmacists in the provision of pharmaceutical services in three countries with distinct healthcare systems, examining how these services operate, the requirements for their delivery, the associated remuneration structures, and the extent of out-of-pocket costs for patients—ultimately analyzing their impact on health outcomes.

## 1. Background

According to the World Health Organization (WHO), chronic diseases or noncommunicable diseases (NCDs) are responsible for approximately 74% of all deaths globally [1,2].

NCDs have a significant impact on both the health and the gross national happiness of populations. Furthermore, they pose a threat to national economies, as they result in a reduced ability of individuals to work, which in turn leads to a decline in national income and productivity [3].

NCDs are influenced by non-modifiable and modifiable risk factors. Non-modifiable risk factors are those which cannot be changed, for example, age or genetics. In contrast, modifiable factors are open to modification, such as high blood pressure, obesity, inactivity and smoking [3].

NCDs frequently have no symptoms or manifest without explicit symptoms during the initial stages [3]. Therefore, the implementation of comprehensive prevention and early detection strategies is essential to ensure timely and appropriate treatment of these conditions, thereby reducing the strain on healthcare systems and mitigating negative economic impacts [1,2,3,4].

Healthcare systems are currently confronted with substantial challenges, which have been further exacerbated by the COVID-19 pandemic [5,6,7]. These challenges stem from multiple factors, including an aging population, a declining healthcare workforce, rapid technological advancements, the emergence of new public health threats, evolving patient expectations, constrained financial resources, chronic underinvestment, and external pressures such as climate change and inflation [5,7].

In response to current challenges, community pharmacies have evolved their role, transitioning from a primary focus on medication dispensing to the provision of comprehensive, patient-centered care and services. This shift emphasizes the delivery of high-quality care aimed at improving health outcomes, enhancing the overall quality of care, and contributing to cost reduction [5].

Pharmacies have a pivotal position within the healthcare system, acting as a primary point of contact with the public and often being the closest healthcare facility to patients [8,9]. Consequently, they possess a significant and undeniable influence on the population’s health [8]. This proximity makes them an ideal setting for implementing straightforward and opportunistic screening programs, often comprising physiological measurements, questionnaires, and risk assessment forms [9].

Pharmacists are highly trained healthcare professionals with in-depth expertise in pharmacotherapy and disease management. Their primary role involves overseeing the dispensing of medications while ensuring their safe and effective use by patients [10]. In recent years, pharmacists’ scope of practice has expanded, with a growing involvement in direct patient care, health promotion, and disease prevention initiatives. These services include administering vaccinations, supporting medication adherence, and conducting comprehensive medication reviews [9,10]. Additionally, pharmacists have great potential in implementing public healthcare programs, undertake measures for the prevention of NCDs, conduct screenings and referrals for individuals exhibiting potential NCD signs and symptoms, and provide support for prescribing practices [9].

In essence, community pharmacies and pharmacists are essential in enhancing accessibility to healthcare, optimizing the benefits derived from medicines, and facilitating the delivery of effective therapy and disease management for patients, thus contributing to the improvement of clinical outcomes [9].

Nevertheless, the availability of pharmacist screening services in community pharmacies may be restricted in certain countries due to, for example, the absence of transparent reimbursement structures or the presence of an overly complex public health legislation [9].

The aim of this study is to examine the delivery of pharmaceutical services, through the pharmacist perspective, in three different countries with distinctive healthcare systems and evaluate some of their health outcomes and impacts. The objective is to provide insights regarding the operational aspects of pharmacies in these countries, including the services provided, the requirements for their provision, the remuneration structures in place, and the level of out-of-pocket expenditure by patients.

## 2. Healthcare Systems

The WHO defines health as “a state of complete physical, mental and social well-being and not merely the absence of disease or infirmity” [11]. Furthermore, the WHO asserts that the achievement of the highest attainable standard of health constitutes a fundamental right of all human beings, and that governments bear the responsibility for the health of their citizens, which can be fulfilled only by the implementation of adequate health and social measures [11].

The concept of a healthcare system has been defined in a number of different ways. In essence, it can be understood as a range of interrelated components, including resources, organizational structures, management practices, economic support, and the delivery of services, which are all oriented towards the primary objective of promoting, restoring, or maintaining health [12,13].

### 2.1. United States of America (USA)

The healthcare system in the United States (U.S.) is notably complex and heterogeneous, comprising a wide range of entities and organizations. In contrast to many other developed nations that operate under a single-payer model or a national pharmaceutical benefits scheme, the U.S. system includes a mix of public and private insurers, nonprofit and for-profit institutions, healthcare organizations, and providers [14]. This system operates under a commercial model, characterized by a multi-payer structure in which private insurance plans are provided by for-profit health insurance companies [15].

Moreover, healthcare in the U.S. is founded upon the principles of demand and supply, with the option for individuals to select from a range of insurance programs, thereby reducing the regulatory role of the state significantly [15].

As of 2023, 68.1% of people aged 18 to 65 in the U.S. had private insurance, 23.0% had public insurance, and 10.9% were uninsured. Private insurance plans encompass those obtained through employers, purchased directly, and purchased through local or community programs, among others [16,17].

In March 2010, President Barack Obama signed the Patient Protection and Affordable Care Act (ACA), a federal law that aims to achieve three primary objectives [18,19,20]:

1. Make affordable health insurance more attainable for a broader demographic by offering financial assistance in the form of subsidies that reduce the costs borne by households with incomes between 100% and 400% of the federal poverty level (FPL).
2. Extend the Medicaid program to include adults with an income below 138% of the FPL, although this has not been implemented in all states.
3. Facilitate the implementation of novel medical care delivery systems with the objective of reducing the overall costs of healthcare.

Furthermore, the ACA also places an emphasis on preventative care, requiring that all plans cover a set of preventive services, such as immunizations and screenings, free of charge to the patient [21,22]. Moreover, the system provides discounts, rebates, and waivers on insurance premiums to businesses that implement workplace wellness programs, thereby promoting preventive care through financial incentives [20].

The ACA mandates that the majority of citizens must enroll in a health insurance plan, unless they qualify for an exemption, such as those based on religious beliefs or financial hardship. Those who fail to comply were subject to a tax penalty that was revoked in 2018; however, some states have retained the obligation to be insured and the penalty for non-compliance [20,21,22,23].

Furthermore, the ACA sets forth regulatory mandates for health insurers, including the requirement to cover individuals with pre-existing conditions at no additional cost and the prohibition of policy cancelations based on the onset of illness in insured individuals [24].

Additionally, the ACA encourages the creation of “Health Insurance Marketplaces”, which are operated by either the federal government or by individual states. These marketplaces provide a platform for individuals to compare and enroll in health insurance plans that are eligible for federal subsidies, in accordance with state and federal regulations [20,25]. A similar platform, the Small Business Health Options Program Marketplace, is also available for small businesses looking to provide health insurance for their employees [25].

The stipulations of the “Health Insurance Marketplace” legislation dictate that insurance policies must adhere to defined requirements, including the provision of coverage for 10 specified health benefits, such as ambulatory patient services, emergency care, and preventive and wellness services, as well as chronic disease management [26,27].

The U.S. federal government offers health insurance plans that are funded by taxes, but only available to certain groups of people. These plans include Medicare for the elderly, Medicaid for those living in poverty or disabled, and the Children’s Health Insurance Program for children younger than 18 who do not qualify for Medicaid [15,28].

The Medicare program has two options for patients to choose: Original Medicare and Medicare Advantage (Part C) [28,29]. The Original Medicare plan comprises two distinct parts: Part A (Hospital Insurance), which covers inpatient care in hospitals, skilled nursing facility care, hospice care, and home healthcare; and Part B (Medical Insurance), which covers doctors and other healthcare providers services, outpatient care, home healthcare, durable medical equipment (such as wheelchairs), and preventive services (such as screenings, vaccines, and yearly wellness visits) [28,29].

A Part D (Drug Coverage) plan may be added to the Original Medicare plan or already exists in a Medicare Advantage plan [28]. Part D plans provide coverage for a wide range of prescription medications commonly used by individuals with Medicare insurance. These drug coverage plans typically include a set of medications, arranged in tiers according to the patient’s out-of-pocket expenses. For instance, a medication in a lower tier usually has a lower cost compared to the patient medication at a higher tier. Additionally, Part D plans are required to offer Medication Therapy Management (MTM) services to members who meet specific criteria or are enrolled in a Drug Management Program [14,15,30].

Medicare Advantage is a Medicare-approved plan from a private insurer which provides an alternative to the Original Medicare plan. It comprises Parts A, B, and usually D, and may offer beneficiaries reduced out-of-pocket costs and supplementary benefits not covered by the original scheme. These include vision, hearing, and dental services [28,29].

It is important to note that the use of both plans is contingent upon the healthcare facility’s acceptance and collaboration with these insurance types [29]. Furthermore, it is possible to hold both a Medicare plan and another form of health insurance [31].

The definition of healthcare providers in the U.S. does not include pharmacists, which precludes them from receiving remuneration from the federal government for the majority of services provided under Medicare Part B [14,28].

The status of a provider would permit pharmacists to utilize fee-for-service health insurance billing codes when providing non-dispensing services [14].

### 2.2. England

The United Kingdom (UK) adheres to a national healthcare plan that is based on the Beveridge model. In fact, Sir William Henry Beveridge himself designed Britain’s National Health Service (NHS). In this system, the financing of healthcare is the responsibility of the government, which operates as a single payer. This is achieved through the collection of taxes. A significant proportion of healthcare facilities are owned by the government, and the healthcare workers are government employees. Although private initiatives are permitted, they are frequently financed by the state. The government, as the sole healthcare payer, is in a position of complete control over the costs of the system [15].

The NHS encompasses a wide range of healthcare services including general practitioner services, pharmacies (which are included as part of primary care), prescriptions, hospitals, mental health services, and services related to COVID-19. Additionally, the NHS provides urgent and emergency care, sexual health services, dental care, optician services, alcohol addiction treatment, online services, and other forms of support [32,33].

The NHS covers the cost of prescription medications, so the patient only pays 9.90 GBP (11.74 EUR) per item. Alternatively, the patient can obtain a “prescription prepayment certificate” that covers medicines during a 3- or 12-month period [34,35,36].

Certain medications are fully reimbursed by the NHS, such as contraceptives and drugs administered to hospital inpatients. Additionally, full coverage is provided for specific patient groups, including individuals under the age of 16, those over 60, and patients with particular medical conditions or low income [34,37,38].

### 2.3. Portugal

In Portugal there are three co-existing and overlapping systems—a national Healthcare Service or “Serviço Nacional de Saúde” (SNS) that is universal and funded by taxes; health insurance based on professional group or company; and private health insurance [39].

The SNS, based on the Beveridge model, provides coverage for a range of healthcare services, including primary care, hospital care, and integrated care. The system is universal (extended to all citizens), but not entirely free of charge. Rather, the price of healthcare is fixed or determined according to the financial circumstances of the patient, although certain groups of people are exempt from charges (pregnant woman, minors, people with low income and their families, etc.) [15,40,41,42,43].

The role of private insurance is mainly supplementary, facilitating access to elective hospital treatment and ambulatory consultations. It is, in rare circumstances, complementary, covering services excluded from the SNS [39].

The government subsidizes a proportion of the cost of certain medications according to a classification into groups—group A (90% of the price is covered), group B (69%), group C (37%), group D (15%) [39,44]. In addition, some medicines (used by highly vulnerable groups of patients—immunomodulators, chronic kidney failure treatment, antivirals for hepatitis C, antiretrovirals for HIV, etc.) are fully covered by the SNS [39].

The Portuguese National Authority for Medicines and Health Products (INFARMED I.P.) is responsible for regulating and supervising the human medicines and health products sector. Its objective is to guarantee access to quality, effective, and safe medicines and health products for health professionals and citizens. Furthermore, this institution oversees the majority of pharmaceutical activities, as pharmacies are obliged to comply with its directives [39,45].

## 3. Community Pharmacies

A community pharmacy is a healthcare establishment that provides pharmaceutical and cognitive services to a defined community [46]. Community pharmacists are considered “the health professionals most accessible to the public and are a cornerstone of primary health care” [47].

Community pharmacies are strategically located within communities, situated on main streets, within supermarkets, and in more rural villages. This allows them to provide health services when other healthcare professionals are unavailable [46].

### 3.1. USA

In the U.S. there are several types of community pharmacy practice settings, such as independent, traditional chain, supermarket, and mass merchant pharmacies. Their main focus is dispensing medications, but some also offer clinical services [14].

In fact, according to a 2023 report, in independent community pharmacies, 71.8% of pharmacists’ time is spent in activities related to medication dispensing. The remaining percentage of time is spent in other patient care services (12.6%), business or organization management (9.8%), education (3.4%), and other activities (2.4%) [48].

The location of a community pharmacy greatly impacts patient’s access to their services [14]. In 2015, an average of 12.7 prescriptions per capita were dispensed by community pharmacies, with over 90% of the population residing within 3.2 km of one. Pharmacy proximity plays a key role in medication adherence, which is also influenced by the availability of accessibility-enhancing services, such as multilingual staff and home delivery options [49].

In the U.S., the ownership and location of a community pharmacy are not subject to regulatory oversight. However, a pharmacy is obliged to have a pharmacist-in-charge as well as a licensed pharmacist present during the pharmacy’s opening hours [15].

### 3.2. England

In the UK, community pharmacy constitutes one of the four pillars of the primary care system, alongside general practice, optical services, and dentistry. While it is most commonly recognized for its role in dispensing and retailing medicines, its scope extends well beyond this function, incorporating a range of NHS and publicly funded services [50].

The community pharmacy operates on a contractor model analogous to that of other primary care providers, such as general practitioners. Under this arrangement, pharmacies are usually independently owned businesses that contract with the NHS to deliver designated services to the local population [50].

The predominant model is that of a commercial establishment that combines retail services with the delivery of selected healthcare services by qualified professionals. Pharmacies act as an easily accessible point-of-care, offering patients guidance and support on medication use for both minor acute conditions and the management of chronic illnesses [50].

A minority of pharmacies operate as online, distance-selling entities that generally do not provide face-to-face services. Instead, they meet their service obligations through alternative delivery methods. A pharmacy may be registered by an individual pharmacist or a partnership composed exclusively of pharmacists. Alternatively, it may be registered by a corporate body, provided a superintendent pharmacist is appointed to supervise all professional activities across the organization [50].

It is mandatory for all pharmacies to have a “responsible pharmacist” in attendance at all times, as a mean of ensuring compliance with safety, legal, and other pertinent requirements. In the event that this individual is not the proprietor, a suitable qualified and registered pharmacist must assume this role [50,51].

The funding of community pharmacies is derived from a number of sources, including remuneration and reimbursement by the NHS as part of their contract work and additional income from both NHS and non-NHS sources. The proportion of revenue derived from the NHS varies considerably across community pharmacies. In general, smaller independent pharmacies rely on NHS income as their primary source of revenue [50].

### 3.3. Portugal

In Portugal, community pharmacies are independent businesses that cooperate with the government and INFARMED I.P. According to Portuguese legislation, their activities are of public health and societal interest, with a central role in promoting the rational use of medicines [43,52].

The number of community pharmacies permitted in each location is limited and subject to strict regulation. Individuals or commercial companies may own pharmacies, as long as they are registered, have less than four pharmacies, are not prescribers and are not involved with the pharmaceutical industry or distribution [39,43,52].

Community pharmacies are authorized to supply a wide range of products, including human and veterinary medicines, natural health products, medical devices, food supplements, special dietary items, plant protection products, cosmetics, childcare goods, and comfort products. In addition, they may provide various pharmaceutical services aimed at promoting patient health and well-being [43,52].

The revenue of community pharmacies is derived primarily from a profit margin applied to the cost of dispensed medications. In the case of prescription medicines, the remuneration system is established by the government [43].

Pharmacists are required to be registered with the Portuguese Pharmacists’ College (Ordem dos Farmacêuticos) in order to perform pharmaceutical activities (e.g., dispensing of medicines, providing pharmaceutical services, interpretating and evaluating medical prescriptions) [53].

The role of technical director is exclusively reserved for pharmacists, who may hold this position at only one community pharmacy and must be registered with INFARMED I.P. The technical director functions independently, adhering to strict deontological and technical standards that ensure and promote the quality and continuous improvement of the services provided. They may be supported by pharmacists, pharmacy technicians, and other appropriately qualified personnel, all of whom operate under the technical director’s supervision and responsibility [43,52].

## 4. Services in Community Pharmacies

The delivery of services through community pharmacies serves as a key approach to providing pharmaceutical care. This care is intended to enhance patients’ overall health and well-being by optimizing medication use and improving health outcomes [15]. A comprehensive overview of the pharmaceutical services presented in this section, the existence of reimbursement structures in each country, and their effects and benefits of each service are summarized in Table 1 and Table 2.

Regarding the U.S., pharmacies may offer a variety of services related to dispensing processes and health-related (e.g., point-of-care-testing) and cognitive services (e.g., medication therapy management). In some states it is mandatory to provide review and counseling services when dispensing medication [54].

Pharmacy practice legislation varies across all states in the U.S., which makes it challenging to spread and scale up pharmacy services [14]. Some pharmacy services can be reimbursed by health systems (e.g., medication therapy management), while others are paid out-of-pocket by the patient (e.g., point-of-care testing) [54]. A 2008 study found that individuals were more inclined to pay for cognitive services if their insurance plan included coverage for a portion of the cost [55].

Moreover, in England, there has been an increased awareness of the unrealized potential in the community pharmacy sector. In recent years, the clinical service offer of community pharmacies has expanded through the provision of a range of services, including Essential, Advanced, National Enhanced, and Locally Commissioned Services. These services are provided by pharmacy contractors and commissioned by the NHS [50,56,57].

- Essential Services: services which have been nationally defined as mandatory for community pharmacies—e.g., dispensing of medicines and medical appliances, disposal of medicines [50].
- Advanced Services: nationally set and specified optional services that any pharmacy may choose to provide, as long as they meet certain minimum requirements—e.g., New Medicine Service [50,56].
- National Enhanced Services: optional services commissioned by the NHS [50,58].
- Locally Commissioned Services—Often considered part of, or similar to, enhanced services. These are commissioned by public bodies to meet specific needs of local populations—for example, needle and syringe exchange programs [50].

Additionally, alongside the services commissioned by the NHS and other public bodies, pharmacies can also provide private services that are not commissioned by public bodies (e.g., travel health advice, vaccinations, and skin treatment services) [50,56]. It is emphasized that, for the provision of most services, pharmacists should notify the NHS and ensure the availability of appropriate consultation rooms, established standard operating procedures, and functional IT systems. Additionally, they should inform the patient’s general practitioner and implement quality assurance mechanisms.

Portuguese community pharmacies may offer a variety of services to patients that should be displayed with the respective prices in an area visible to the patient [43,59]. It is possible for pharmacies to enter into agreements with other professionals in order to provide services such as podiatry and nutrition consultations. The services should be provided by suitably qualified professionals, obey legal requirements, take place in adequate installations, and follow the INFARMED’s instructions, if available [60].

Most services offered by pharmacies are subject to free pricing and are paid for out-of-pocket by patients, resulting in considerable price variability. Portuguese law allows pharmacies to deliver public health intervention services, including primary healthcare programs, participation in health technology assessments, needle exchange initiatives, monitoring of treatment adherence, and the dispensing of medicines traditionally provided by hospital pharmacies. However, only a limited number of these services—such as the needle and syringe exchange program—are offered free of charge to patients and reimbursed to pharmacies [15,43,61].

A 2017 study reported that “community pharmacies services in Portugal provide a gain in Quality of Life (QoL) of 8.3% and an economic value of EUR 879.6 million, including EUR 342.1 million in non-remunerated pharmaceutical services and EUR 448.1 million in avoided expense with health resource consumption. Potential future community pharmacy services may provide an additional increase of 6.9% in QoL and be associated with an economic value of 144.8 million EUR [62].

The challenges associated with integrating community pharmacies with primary care are essentially related to the nature of community pharmacies as private businesses and the government’s reluctance to collaborate with the private sector in the healthcare area. Another significant challenge pertains to the accessibility of patient health records and the incorporation of pertinent information to enable pharmacists to access and update these records with relevant interventions [43].

The obstacle pertaining to the lack of access to patient information may potentially be eliminated in the future, assuming the implementation of the European Health Data Space (EHDS). The EHDS is defined as “a health-specific data sharing framework establishing clear rules, common standards and practices, digital infrastructures and a governance framework for the use of electronic health data by patients and for research, innovation, policy making, patient safety, statistics or regulatory purposes”. The proposed data space would facilitate access to patient information by health professionals from disparate EU countries, thereby enhancing the quality of treatment and diagnosis, and facilitating more informed decision-making [63].

### 4.1. Dispensing Medication

Dispensing prescription medication remains the core activity of pharmacists worldwide. This process also offers a valuable opportunity for pharmacists to engage with patients about their medications, helping to optimize medicine use, improve health outcomes, and support better disease management and control [15].

#### 4.1.1. USA

Unlike Europe, where pharmacies dispense medicines in individual boxes sold by the pharmaceutical industry, U.S. pharmacies typically dispense medicines in prescription bottles from bulk containers. This means that usually pharmacy technicians are primarily responsible for filling and labeling medicines, which are subsequently checked by the pharmacist [14].

The majority of states mandate that a patient drug profile be kept at the pharmacy and reviewed prior to dispensing a prescription. This review serves to identify potential drug interactions and contraindications. Additionally, at the time of the dispensing, pharmacists are required to inform patients on how to use the medication and provide counseling to those who are receiving new prescriptions [54].

In 2017, the total expenditure on prescription drugs in the United States of America was USD 333 billion, with 82% of this amount supported by the three primary sources of payment within the USA health system: private health insurance (42%), Medicare (30%), and Medicaid (10%). Patient out-of-pocket costs constituted 14% of the total expenditure on retail drugs [64].

Pharmacies generate revenue by charging a markup on the price of drugs and a dispensing fee. Insurance providers usually reimburse pharmacies for the drug cost, using an estimated average price, while pharmacies can usually purchase drugs at a lower price than the average wholesale price (AWP) [54].

The maximum allowable fee for brand name drugs is calculated at 85% of the AWP plus a USD 4 (EUR 3.67) dispensing fee. For generic drugs and non-drug items, the maximum allowable fee is calculated at 70% of the AWP plus a USD 4 (EUR 3.67) dispensing fee [65].

##### Medication Synchronization (USA)

This consists of aligning the patient’s medication refill dates, so that patients only come to the pharmacy once a month [14]. Appointment-Based Medication Synchronization (ABMS) is a system that can be implemented in any pharmacy setting; it improves both patient adherence and pharmacy operations efficiency [66].

In this system the patient has a designated day to pick up all medications prescriptions. The pharmacy calls the patient beforehand to identify changes to the medications and confirm each prescription to be refilled, which allows the pharmacy to manage potential issues before the patients arrive to pick up their medicines [14,66].

This system enables a shift in focus, allowing pharmacists to conduct a comprehensive monthly review of all medications. As a result, the process of filling or refilling prescriptions becomes more rational, proactive, and patient-centered [66].

A quasi-experimental study comparing patients enrolled in an Appointment-Based Medication Synchronization (ABMS) program to control patients demonstrated that the ABMS program significantly improved medication adherence. Patients in the program had 3.4 to 6.1 times greater odds of adherence, while control patients were 52% to 73% more likely to discontinue their chronic medications over the course of one year [67].

This service is not reimbursed, and some pharmacies focus exclusively on the alignment of refill dates, while others conduct an ABMS where patients meet with the pharmacist to discuss their medications, any adherence issues, or receive reimbursed MTM services [14,66].

#### 4.1.2. England

The dispensing of medications by pharmacies is conducted in accordance with either a digital (Electronic Prescription Service) or paper prescription. Pharmacies are obliged to maintain a comprehensive record of all medicines dispensed, along with any interventions they deem to be significant [68,69]. All prescriptions attract a fee of GBP 1.27 (EUR 1.51) for the dispensing of the prescription. Furthermore, additional fees may be applicable, depending on the nature of the medication, appliance, or service in question [55].

In most cases, the pharmacy reimbursement is contingent upon the dispensing of the precise quantity prescribed [70]. In certain instances, a pharmacist may be required to divide an original medication package, in order to dispense the prescribed quantity. In these cases, reimbursements are also provided to account for the additional costs associated with obtaining supplementary packaging and patient information leaflets [71,72].

##### New Medicine Service

The New Medicines Service (NMS) offers guidance to individuals with specific long-term conditions (such as asthma and COPD, type 2 diabetes, hypertension, epilepsy, and Parkinson’s disease) when initiating treatment for an eligible medication. To provide this service, pharmacists require additional certification [15,73,74,75].

Additionally, this service presents an opportunity to promote lifestyle changes or other non-pharmacological interventions in order to enhance well-being in individuals with these long-term conditions. The implementation of NMS was associated with an increase in patient medication adherence compared to standard practice. This resulted in a greater overall health benefit at a reduced cost [15,74,75].

The NMS can be conducted in either a pharmacy setting or remotely, provided that the conversation is conducted in private. The service comprises three distinct sections [73,74,75]:

1. Patient Engagement: Patients may be recruited via a prescriber, healthcare professional referral, or may also be opportunistically identified by the pharmacist.
2. Intervention: The pharmacist will evaluate the patient’s adherence to the medication(s), identify any potential issues, and determine the necessity for additional information and support. They will then provide counsel and assistance to the patient and agree on a course of action.
3. Follow Up: The pharmacist will evaluate the patient’s compliance with prescribed medications, identify potential issues, and determine the patient’s need for additional guidance and resources.

##### Repeat Prescription

In cases where patients are required to take medication on a regular basis (due to long term conditions such as diabetes and hypertension), their GP may issue them a repeated prescription. This allows the patient to obtain their medication as needed without having to consult their GP for each refill [76,77,78].

The service is designed to save time for both general practitioners and patients, minimize waste by ensuring that only necessary medicines and appliances are dispensed, and enhance convenience and access to prescriptions. Additionally, it allows community pharmacists to take on a more proactive role in ensuring the safe and consistent supply of regular medications. Through the repeat prescription system, prescribers can electronically authorize and issue a batch of prescription items for a period of up to 12 months [78].

The prescriber also determines the interval between repeats, taking into account the clinical needs of the patient. The default interval is 28 days, which gives the patient access to a repeated dispensing service every 28 days and they receive their prescribed medications for that period [78].

In the repeat dispensing service, pharmacy teams will do the efollowing [77,78]:

- Dispense repeat dispensing prescriptions issued by a GP.
- Ensure that each repeat supply is necessary.
- Seek to establish whether there are any reasons why the patient should be referred back to their GP.

To support timely preparation of medications before a patient visits the pharmacy, the designated pharmacy automatically receives the next repeat dispensing batch seven days before the expected end of the current supply [77,78].

#### 4.1.3. Portugal

The dispensing of reimbursed prescription medicines is the major source of income in Portuguese pharmacies, followed by Over the Counter (OTC) and pharmacy-only medicines, as well as other products [79].

When dispensing prescription medication, the pharmacist must review both the prescription, whether electronic or manual, and the patient’s information to identify any potential errors. Medications should then be dispensed in accordance with the prescription. Additionally, the pharmacy is required to stock at least three of the five lowest-priced medicines that contain the same active substance, dosage, and pharmaceutical form, allowing the patient to choose their preferred brand [59,80,81].

When dispensing non-prescription medication, the pharmacist should analyze the patient’s necessities and characteristics, as well as the medication itself to make sure it is adequate, safe and it is delivered with proper advice, thus promoting a rational use of the medication [59,82].

##### Proximity Dispensing of Medicines

The objective of this service is to provide patients with the option of accessing medicines and other health products that are exclusively for hospital use in a community pharmacy. This ensures proximity, maintains the safety and pharmaceutical monitoring of the treatment, and provides traceability of the dispensing process [83,84].

Proximity dispensing services were developed during the COVID-19 pandemic, when it was particularly important for patients to avoid risk of infection, especially those vulnerable and their family members. Furthermore, it enabled the continuity of therapeutic benefits and health outcomes for those undergoing treatment, prevented interruptions in adherence, and permitted the reallocation of hospital resources [84,85].

The hospital pharmacist plays a central role in this process, ensuring the integration of all relevant information pertaining to the processed data, making it imperative that both hospital and community pharmacies adhere to the legislative requirements in order to provide this service [84,85].

The SNS is the sole sponsor of the proximity dispensing of medicines, providing a remuneration of EUR 11.96 per dispensing. The payment is distributed as follows: 57.07% to the pharmacy, 25.37% to the distributor, and 17.56% to the entity responsible for the central warehouse [86].

##### Therapeutic Renewal

Similarly, to the Repeat Prescription Service, the Therapeutic Renewal service enables patients with chronic conditions to renew their prescriptions at a community pharmacy, without the necessity of accessing primary healthcare. Furthermore, doctors are allowed to electronically prescribe certain medications for a period of 12 months, although pharmacists are only able to dispense such medications for up to two months. Prior to dispensing, the pharmacist should access whether the patient still requires the medication or if there are potential contraindications for its use [84,87,88].

Portuguese pharmacists are able to access data on prescriptions and dispensing of medicines and health products over the last 12 months. Additionally, they are also able to write “therapeutic notes”, which are intended to inform the patient’s GP of a range of issues, such as instances of non-adherence, interactions between medications, adverse effects of the medications, difficulties in achieving therapeutic goals, and so forth. Additionally, this system also enables the GP to respond to the pharmacist’s notes [87,88].

The renewal of therapy at pharmacies has been found to enhance the patient experience, facilitate access to care, improve adherence to treatment regimens, and empower patients to participate in their own clinical decision-making. Furthermore, it releases resources and alleviates the burden on the SNS [84,87].

### 4.2. Immunizations

Community pharmacists have the potential to contribute to the development of effective vaccination strategies, thereby safeguarding the public’s health and ensuring the long-term sustainability of the healthcare system. Community pharmacists, due to their proximity to the public, are well-positioned to implement measures aimed at reducing the burden of communicable and vaccine-preventable diseases, even though the typical duties of a pharmacist (dissemination of information, provision of advice, referral of patients to appropriate care, administration of treatments, undertaking of preventive actions) already contribute to the reduction of those diseases [89].

A literature review reveals a correlation between community pharmacies’ involvement concerning administration of vaccines and several beneficial outcomes. These outcomes include increased immunization rates, convenience of vaccination, health access and equality, and positive economic and health effects [90,91,92,93,94].

#### 4.2.1. USA

U.S. pharmacists can administer immunizations in community pharmacies across all the country. In fact, in 2019, 90% of community pharmacists reported administering vaccines [95]. Moreover, in 2022, most independent community pharmacists spent 1–10 h/week administering vaccines, with most chain community pharmacist spending over 11 h/week. Nevertheless, it is important to note that there are restrictions that vary from state to state, limiting the types of vaccines that can be administered by pharmacists and the characteristics that must be met for individuals to qualify to receive specific vaccines (e.g., age) [14].

A 2016 survey of pharmacy-based immunization services, that accounted for 292 USA pharmacies, reported that the most commonly administered vaccine types were Influenza (provided by 96.1% of pharmacies, 484 average doses/pharmacy), Pneumococcal 13-valent conjugate (90.0%, 55 doses/pharmacy), Herpes Zoster (91.4%, 41 doses/pharmacy), and Pneumococcal polysaccharide (88.6%, 39 doses/pharmacy). This service was also associated with an increase in the likelihood of immunization for influenza and pneumococcal diseases [96].

In order to bill this service, pharmacies need to deal with the appropriate payers (e.g., Medicare, third-party medical plans, etc.). The payment includes the vaccine itself and an administration fee, usually out-of-pocket for the patient, that can vary between USD 15 and 40.70 (EUR 14–37.36) [97,98]. The average cost of vaccination in a pharmacy was shown to be lower when compared to other medical settings [99].

#### 4.2.2. England

The NHS vaccination strategy for the flu and COVID-19 vaccines includes the provision of vaccinations in pharmacies, which are free for eligible patients. Patients may utilize the National Booking Service to schedule appointments for influenza and COVID-19 vaccines in pharmacies via the Internet [100].

The provision of influenza vaccinations by pharmacies is regarded as an advanced service, with the 2024/25 season fee set at GBP 9.58 (EUR 11.36). This is paid to pharmacy owners, who also receive reimbursement for the basic price of the vaccine. On the other hand, the administration of the COVID-19 vaccine is classified as a national enhanced service, for which pharmacy owners are compensated at a rate of GBP 7.54 (EUR 8.94) (or GBP 10 (EUR 11.86) if the patient is housebound) per vaccine administered [101].

Furthermore, pharmacies are able to provide travel healthcare services, which may include travel vaccines that may not be covered by the NHS and necessitate out-of-pocket expenses for the patient [102].

#### 4.2.3. Portugal

Portuguese pharmacies are permitted to provide administration of injectable medication and vaccination services for vaccines not included in the national vaccination plan (e.g., vaccines for varicella-zoster virus, hepatitis A, rotavirus and respiratory syncytial virus). In order for these vaccines to be administered, patients must present a valid prescription, and pharmacies must employ certified pharmacists who are competent in the administration of vaccines and other injectable medications. Additionally, pharmacies must adhere to the other requirements within the legislation (e.g., having adequate installations, equipment, and materials) [59,103,104,105]. The pricing of this service is determined by each pharmacy.

Pharmacies are permitted to participate in the SNS’s Seasonal Vaccination Campaign, whereby pharmacists administer a flu and COVID-19 vaccine to certain groups of people free of charge. It is a prerequisite that pharmacists have completed the required training, and that pharmacies comply with all other relevant demands. The vaccines are provided to pharmacies by the government, and the pharmacy is remunerated with a fee of EUR 2.5 per vaccine after registering the service [106,107].

A recent study suggested that the 2023/2024 Seasonal Vaccination Campaign resulted in a potential saving of approximately EUR 2.4 million for patients and saved 310,000 h of work for SNS’s health professionals. Furthermore, the study indicates that vaccination at pharmacies enhanced accessibility and patient satisfaction, potentially enabling some patients to save financial resources [108].

### 4.3. Medication Review and Medication Reconciliation

Medication Review and Reconciliation processes can be defined as a systematic approach to optimizing patient safety, effectiveness, and efficiency of therapies on different levels. Whereas the objective of a Medication Reconciliation is to obtain a complete and accurate list of the patient’s current medications with the purpose of identifying and resolving discrepancies, a Medication Review focuses on evaluating the patient’s medications with the goal of detecting and solving drug-related problems (DRPs). Therefore, it is reasonable to consider that a Medication Review may need a Medication Reconciliation as a preliminary step [15,109,110,111].

The availability of information is a significant factor influencing the ability to detect DRPs. The use of a greater number of sources of information increases the number and types of DRP that can be identified. Indeed, medication reviews are classified according to the type and source of available information. For instance, a medication review based solely on pharmacy claims data or pharmacy medication histories may identify issues such as excessive dosage, drug–drug interactions, and therapeutic duplication. However, a more comprehensive medication review that incorporates medication history, clinical data, and information from a patient interview may uncover additional DRPs, including the use of a drug without an indication or an inappropriate dosage form. It is crucial to emphasize that a patient interview is a vital component in addressing the patient’s drug-related needs [15].

#### 4.3.1. Medication Review

According to the Pharmaceutical Care Network Europe, “Medication Review is a structured evaluation of a patient’s medicines with the aim of optimizing medicines use and improving health outcomes. This entails detecting DRPs and recommending interventions” [112].

Medication Reviews are classified in different types according to the level of information available (medication history, clinical data), the patient’s involvement (patient interview), and the level of multidisciplinary collaboration. Medication Review’s relevance is increasing around the world, mostly due to the increase in polypharmacy. Although no consensual understanding exists, polypharmacy is commonly defined as the concurrent use of five or more medications [113,114]. In both settings, community and hospital, Medication Review is considered a useful tool to detect and resolve polypharmacy problems [15,110,115].

Medication Use Review (MUR) is a type of Medication Review based on medication history and patient information (patient interview), which highlights drug interactions, side-effects, unusual dosages, adherence, and effectiveness issues [115,116].

Medication Review includes 4 core elements [15,115,117]:

1. Data collection:
  - Best Possible Medication History (BPMH): The patient’s Personal Medication Record (including prescription and OTC medications, herbal products, and supplements). It is recommended that more than one source of information is used.
  - Patient Interview: As a Medication Therapy Review, involving a systematic process of collecting patient-specific information, accessing the validity of the medication history, and highlighting medication-related issues. It may include information on allergies and previous adverse drug events. The Brown Bag Review may be a more pragmatic and useful approach to gain a better understanding of the patient’s medication experience.
2. Detection and evaluation of DRPs: Should consider objective and subjective data, using a systematic, reproducible approach with standardized tools and methods—implicit (Medication Appropriateness Index) and explicit (Beer’s Criteria, STOPP & START Criteria) tools.
3. Agreement on interventions: Interventions and solutions for the identified DRPs are defined and proposed.
4. Documentation and Follow-up: All steps of the service should be documented in a standardized manner. A record should be kept of all DRPs identified, the recommendations, and whether follow-up is necessary.

All healthcare professionals with the appropriate knowledge can conduct a Medication Review. However, the prevalence and complexity of polypharmacy means that the professional responsible must have extensive knowledge of both medications and patient-related outcomes if the goals of optimizing medication use and improving health outcomes are to be achieved. This puts pharmacists and physicians in a favorable position [15,115].

Unfortunately, in many countries within the community setting, it is difficult or nearly impossible to obtain the most complete or detailed types of Medication Reviews because of the limited or difficult access to clinical data. It is recommended that certain groups of patients who are most likely to benefit from a Medication Review (over 65 years of age, polypharmacy, etc.) receive one at least once a year or whenever there is a transition of care [15,110,115].

Lastly, it is important to note that Medication Reviews contribute to decreasing medication errors, optimizing medication use, and improving health outcomes, all with a positive economic impact [110].

#### 4.3.2. Medication Reconciliation

Medication Reconciliation is the formal systematic process by which healthcare professionals access all prescribed and currently used medicines (including OTC and supplements), compare them, identify any discrepancies, and document any changes, resulting in a complete and accurate medication list. Medication Reconciliation usually takes place at transitions of care, as these are critical points where medication discrepancies often occur, in either community or hospital settings [15,109,110,111].

Medication discrepancies (particularly intentional undocumented and unintentional discrepancies) may lead to medication errors that can harm patients and/or hospital admission, thus increasing healthcare costs [15,109,110].

In fact, according to a systematic review and meta-analysis, around “1 in 30 patients is exposed to preventable medication harm in medical care, and more than ¼ of this harm is considered severe or life-threatening” [118].

Medication Reconciliation usually involves the following steps [15,109,119]:

1. Creating the BPMH.
2. Comparing the BPMH with the prescribed medication and identifying discrepancies.
3. Resolving and classifying discrepancies, taking and documenting the appropriate corrective measures.
4. Compiling a new best possible medication list.
5. Counseling the patient and, if needed, scheduling a follow-up appointment and/or checking the need for a Medication Review.

Challenges in carrying out a proper Medication Reconciliation include difficulties in obtaining a complete BPMH, the inability to access relevant information (discharge or hospitalization information), the lack of appropriate reimbursement, and issues related to documentation systems [15,109].

Medication Reconciliation is a key contributor to patient safety through the identification of intentional and unintentional medication discrepancies at transitions of care. Although other healthcare professionals are capable of performing this service, pharmacists have the skills, knowledge and experience to quickly and more effectively detect and resolve inappropriate medication changes and ultimately prevent medication errors and harm [15,109,120].

#### 4.3.3. USA

Medication Therapy Management (MTM) encompasses a range of healthcare services delivered by pharmacists, including medication reviews [109]. Developed in collaboration with licensed and practicing pharmacists and physicians, MTM services may be provided by pharmacists or other qualified healthcare professionals. The primary goal is to improve clinical outcomes by optimizing medication use and minimizing the risk of adverse drug events [121,122].

Some Medicare part D beneficiaries that meet pre-specified criteria (including multiple chronic diseases, taking multiple Part D medications and being likely to exceed predefined medication costs) are eligible to enroll in MTM programs free of charge [14,123].

MTM programs consist of a Comprehensive Medication Review (similar to Medication Review) annually or a Targeted Medication Review (similar to medication Reconciliation) quarterly [14,121].

Reimbursement to pharmacists is provided by the Centers for Medicare & Medicaid Services (CMS) through three different codes [14,124]:

- Initial 15 min for face-to-face assessment charge USD 50 (EUR 46) and is covered once a year per provider per beneficiary.
- Follow up assessments charge USD 25 (EUR 23) at 15 min increments, and providers can bill up to 7 times a year.
- Additional 15 min increments can be billed up to four times per provider per beneficiary per date of service at a rate of USD 10 (EUR 9).

Limiting factors of MTM in the U.S. include deficient standardization for documentation and billing, and lack of access to the electronic health record [14]. A retrospective cohort study involving patients with Medicare Part D coverage who received MTM services concluded that these interventions led to significant reductions in healthcare utilization and improvements in medication adherence [121].

MTM has been associated with improved clinical outcomes, including better cardiovascular health, lower blood cholesterol levels, and improved hemoglobin A1C. It is also linked to positive changes in patient behavior, such as increased knowledge and satisfaction, as well as enhanced medication adherence. Additionally, MTM contributes to a reduction in adverse drug reactions and improvements in both quality of care and quality of life. Emerging evidence also suggests that medication reviews may have beneficial effects on health equity and economic outcomes [125].

#### 4.3.4. England

##### NHS Discharge Medicines Service

The Discharge Medicines Service is a vital initiative that supports patients undergoing transitions in care, particularly those who meet specific criteria or are prescribed medications associated with a higher risk of harm or hospital readmission. Eligible patients are referred to a community pharmacy for a Medication Reconciliation, along with detailed information about any changes made to their medications during their hospital stay. This enables community pharmacy contractors to collaborate effectively in improving patient outcomes, optimizing medication use, preventing medication-related harm during care transitions, reducing hospital readmissions, and enhancing patients’ understanding of their treatment [126,127,128].

The NHS England’s Medicines Safety Improvement Programme has recognized this service as a key contributor to enhancing patient safety during transitions of care, as demonstrated by reductions in hospital readmissions. To implement the service effectively, pharmacy contractors must ensure the availability of private consultation rooms that maintain patient confidentiality, establish a clear standard operating procedure, and confirm that all pharmacy professionals involved are adequately trained and competent to deliver the service [126,127,128].

The service is divided into three stages [127,128]:

- Stage 1: The community pharmacy receives a discharge referral; a review is undertaken by a community pharmacist.
- Stage 2: The community pharmacy receives the first prescription following discharge and will ensure that the prescribed medications align with the changes made during the hospital admission.
- Stage 3: The community pharmacist discusses the medication regimen with the patient and ensures that the patient understands it. This discussion offers a valuable chance to identify and address any adherence issues, clinical concerns, potential questions, or unmet needs the patient may have.

At the start of the service implementation, a set-up fee of GBP 400 (approximately EUR 474) was provided to cover initial preparation costs. Pharmacy owners delivering the full service are remunerated at a rate of GBP 35 (EUR 41). If only part of the service is delivered, contractors are compensated proportionally: GBP 12 (EUR 14) for Stage 1, GBP 11 (EUR 13) for Stage 2, and GBP 12 (EUR 14) for Stage 3 [56,126].

#### 4.3.5. Portugal

##### Medication Review

A Clinical Medication Review is defined as a structured process in which an individual’s pharmacological treatment is evaluated in relation to their overall health, personal circumstances, and specific medical conditions. This review should involve the patient and be conducted with full access to their clinical records and relevant laboratory data. A Medication Review may be initiated by the patient’s physician or another healthcare professional, requested by the patient, or recommended by the pharmacist, particularly when the patient is aged 65 or older, taking multiple medications, has low health literacy, reports difficulties with medication use, or frequently accesses healthcare services [129].

Medication Reviews should be conducted in accordance with the protocol established by the Portuguese Pharmacists’ College. This protocol outlines that the process may involve an initial patient interview, followed by the review itself (performed by a minimum of two pharmacists in collaboration with other healthcare professionals) and appropriate documentation of the findings and outcomes [129].

This service is paid out-of-pocket by the patient, and it should be conducted as an initial process of the individual medication preparation protocol [130].

##### Medication Reconciliation

Medication Reconciliation is defined as the process of obtaining and comparing the complete and exact list of pre-hospital medication with the hospital medical treatment plan (issued at the time of admission, during transfer, and upon discharge) to detect discrepancies in the medication prescribed. Medication Reconciliation services are more prevalent in hospital settings, although community pharmacists may also collaborate with other healthcare professionals providing this service [131,132].

### 4.4. Medication Adherence

Approximately 30% to 50% of medications prescribed to adults with chronic illness are not taken as prescribed. It is estimated that in the U.S. alone, medication non-adherence is associated with 125,000 deaths, 10% of hospitalizations, and USD 100 billion in healthcare services annually [133]. There are many strategies and programs that aim to improve medication adherence and can be implemented in a community pharmacy context.

#### Medication Packaging

A systematic review and meta-analysis concluded that packaging interventions significantly improve medication adherence and found that interventions were most effective when they used blister packs and also delivered in pharmacies [134].

A study involving 92 patients aged 65 and older enrolled in an Adherence Packaging Program (which included medication synchronization, monthly home delivery of packaged medications, and monthly medication reconciliation and review by clinical pharmacists) found that pharmacists prevented an average of 1.87 medication errors per patient [135].

##### USA

This service is not reimbursed by CMS or prescription insurance plans. Some pharmacies may offer this service as part of a medication synchronization service; others may charge an out-of-pocket fee [14].

##### England

Pharmacies offer more adherence services independent from the NHS, such as medication packaging in blister packs, which may be paid for out-of-pocket by patients or may be a free service provided by the pharmacy, although the patient may have to pay a dispensing fee. There is also an NHS recommended service that reminds patients to take their medications and delivers the medications to patients’ homes, through an app [136,137,138,139].

##### Portugal

The PIM (Individualized Medication Preparation) is a service that is paid out-of-pocket by the patient, where the pharmacist organizes the patient’s solid oral medications according to the prescribed regimen in a multi-compartment device, which is then sealed and can be discarded after its use. It is essential that the service adheres to an official protocol. The referenced protocol stipulates that a medication review should be conducted at the start of the service and whenever there are changes to the patient’s therapeutic regimen [130].

PIMs are designed for patients who take more than four medications and have difficulties adhering to their treatment plan. The objective is to help patients follow their therapeutic regimen and promote the rational use of medication [84]. The pricing of this service is determined by each pharmacy.

### 4.5. Hormonal Contraception

The utilization of contraception enables individuals to determine the number of desired children and regulate the frequency of their pregnancies. The prevention of unwanted pregnancies has demonstrated a reduction in the incidence of maternal illness and deaths related to pregnancy, as it allows postponing or avoiding pregnancies among women who have elevated risk of adverse health effects associated with childbearing. The reduction in unintended pregnancies resulted in a decrease in the number of abortions and the HIV transmission from mothers to newborns [140,141].

Moreover, this enables women empowerment by creating a positive impact on their education and expanding opportunities for them to engage fully in society. Hormonal contraceptives encompass a wide range of methods, including oral pills, implants, patches, and vaginal rings. These methods release small amounts of one or more hormones, which prevent ovulation [140,142,143].

#### 4.5.1. USA

As of April 2024, 29 U.S. states permit pharmacists to prescribe hormonal contraceptives. In some states, this prescribing authority requires a collaborative practice agreement, while others allow pharmacists to prescribe independently. Prescribing protocols vary by state but commonly include requirements such as assessing the patient’s blood pressure and completing a self-screening questionnaire or risk assessment. States also differ in terms of age restrictions (either no minimum age or limited to individuals over 18), the types of contraceptive formulations permitted (oral, patch, ring, or injection), and the training requirements pharmacists must fulfill [14,144,145].

Reimbursement may exist or not, and the pharmacy may charge a service fee (typically USD 30–50 (EUR 28–46)) to the patient. The lack of reimbursement of the pharmacist’s time consists of an important barrier that results in some pharmacies opting not providing birth control services even if their state allows it [14,144,145,146].

A 2020 systematic review reported that most pharmacists included in the studies were interested in participating in pharmacist-prescribed contraception services as well as most patients who supported pharmacist-prescribed contraception. Both groups identified several benefits that the service could offer, such as improved patient access, reduction in unintended pregnancies, and professional development for pharmacists. Barriers to implement this service, according to pharmacists, included the need for additional training, payment, time and resource constraints, liability, and patient health concerns [146].

A 2019 study found that expanding access to contraception through pharmacist prescribing could contribute to reducing unintended pregnancies, highlighting pharmacists as well-positioned to fulfill this role. However, notable barriers persist for both pharmacists and patients [147]. Additionally, a 2020 cross-sectional survey revealed that most women opted to obtain contraception from a pharmacist because no appointment was necessary or their previous prescription had expired, with the vast majority reporting high satisfaction with the service [148]. Finally, a 2021 one-year prospective cohort study found no significant difference in the use of effective contraception between women who received their initial prescription from a pharmacist and those who obtained it from a clinician [149].

#### 4.5.2. England

##### NHS Pharmacy Contraception Service

The pharmacy contraception service (PCS) is an advance service provided by community pharmacies that enables pharmacists to initiate and continue the provision of Oral Contraception (OC) (progesterone-only and combination pills), according to Patient Group Directions (PGD) [150,151,152].

Contraception services are free and confidential on the NHS. The service is designed to give patients who meet certain PGD criteria a greater choice and accessibility when considering starting or continuing their current form of OC, and provide additional capacity in primary care and sexual health clinics. A person may self-refer or be referred to a participating pharmacy or the pharmacy itself may identify a suitable person. The PCS is carried out by a pharmacist who has completed additional mandatory training, in accordance with the PGDs. It consists of a confidential consultation based on shared decision-making principles [150,152].

To provide this service, the pharmacy must have a standard operating procedure in place, a suitable consultation room, appropriate equipment (in case it is necessary to measure blood pressure or calculate BMI), and an adequate IT system. If the person consents to the outcome of the consultation being shared with their GP, a notification could be sent accordingly. If the person is not registered or does not agree to share the information, the consultation can still take place, and no further notification is required [150,152].

There is a set-up fee of GBP 900 (EUR 1067), payable in installments: GBP 400 (EUR 474) when the service is registered + GBP 250 (EUR 296) after the first five appointments + GBP 250 (EUR 296) for another five appointments. The fee for each appointment is GBP 18 (EUR 21). The OC supplied is reimbursed in accordance with the Drug Tariff determination [150,152,153].

NHS community pharmacies are widely recognized as accessible and convenient locations where individuals can receive advice and support in managing their contraceptive needs. The PCS underscores the vital role that community pharmacy teams play in reducing health inequalities by improving access to healthcare within their communities [150,151].

#### 4.5.3. Portugal

In Portugal, the acquisition of hormonal contraception by a patient is contingent upon the presentation of a prescription. Pharmacies can only dispense emergency contraceptives (EC). Pharmacies are authorized to dispense the three- (Levonorgestrel) or five-day (Ulipristal) EC. The five-day EC is available exclusively in pharmacies and may only be dispensed by a pharmacist according to the INFARMED’s protocol. Dispensing emergency contraception constitutes an opportunity for pharmacists to provide contraceptive counseling and health education [154].

### 4.6. Point-of-Care Testing

Point-of-care testing (POCT) refers to laboratory testing conducted near the patient care site, such as in a community pharmacy. POCT can be a valuable tool for monitoring health status, assessing disease risk, and diagnosing certain conditions, while also promoting greater patient involvement in the self-management of their health [14,155].

#### 4.6.1. USA

In order to perform POCT, pharmacies require certain certifications and can only perform Clinical Laboratory Improvement Amendment (CLIA)-waived tests (designed for screening, monitoring, or diagnosis outside of a laboratory setting). Some states do not allow pharmacies to perform any tests, or the legislation is unclear [156].

Some examples of POCT in pharmacies include blood HbA1C and glucose, blood pressure, INR, cholesterol, influenza, HIV, hepatitis C, and COVID-19 tests. This service can be reimbursed by a third party or charged directly to the patient. The prices vary depending on supplier and type of test, from around USD 5 to 50 (EUR 5–46) [14,155,156,157].

After testing, pharmacists should, if necessary, report the results, prescribe medication, provide counseling, or refer to a specialist [14,158].

#### 4.6.2. England

Community pharmacies can provide testing and clinical services which involve timely and convenient monitoring for the patient as part of a continued care. NHS-commissioned POCT services include blood pressure monitoring and checks (Hypertension Case-Finding Service), Urinalysis, Chlamydia screening, Carbon monoxide monitoring, COVID-19 rapid antigen testing, Blood glucose measurements, Oxygen saturation, Peak flow measurements, and Hepatitis C Antibody Testing [159,160].

Point-of-care testing (POCT) is often provided free of charge to eligible patients. For example, through the NHS Community Pharmacy Hypertension Case-Finding Service or as part of other NHS initiatives such as Pharmacy First. Test results should be recorded in the patient’s medical record. A designated pharmacy lead is responsible for overseeing the correct implementation of POCT procedures, ensuring that a clinical governance framework is in place and actively monitored, and that all staff using POCT devices adhere to the established standard operating procedures (SOPs) [160,161,162,163].

##### Lateral-Flow-Device Service

The NHS offers COVID-19 treatment to infected people who are at risk of becoming seriously ill. A lateral flow device (LFD) test is used to confirm COVID-19 infection, allowing eligible patients access to appropriate treatment. The LFD service is an advanced, walk-in service, where eligible patients can collect one box of 5 LFD tests from a designated community pharmacy. Patients are encouraged to obtain the tests before they develop symptoms [164,165,166].

Community pharmacies are ideally placed within the community to provide local, rapid access to LFD tests. Pharmacies are reimbursed for the tests in accordance with the Drug Tariff. In addition, for each completed supply of COVID-19 LFD test kits, there is a service fee of GBP 4 (EUR 4.74) [165,166].

##### Hypertension Case-Finding Service

In 2019, 33% of deaths worldwide were due to heart disease. Ischemic heart disease was the leading cause of death (16%), followed by stroke (11%). Worldwide, high blood pressure is estimated to cause approximately 12.8% of the total of all deaths. Hypertension is a major risk factor for coronary heart disease, as well as for both ischemic and hemorrhagic stroke, making it one of the leading causes of premature death worldwide [167,168].

An estimated 1.28 billion adults aged 30–79 years worldwide have hypertension. Approximately 46% of these adults are unaware of their condition, and approximately 21% have it under control [168]. Subsequently, cardiovascular disease (CVD) represents one of the principal causes of premature mortality in England, with hypertension being the greatest risk factor for CVD and a very significant risk factor for premature death and disability in England [161,162,163].

The identification of hypertension at an early stage is of utmost importance. Community pharmacies have a key position in the detection and subsequent treatment of hypertension and CVD, with the potential to enhance outcomes and relieve the burden on GPs [162,163].

The Hypertension Case-Finding Service is an advanced service that aims to identify hypertension cases in people aged over 40 (or under 40, at the discretion of the pharmacy staff). This is achieved through a blood pressure check and ambulatory blood pressure monitoring (ABPM), if appropriate. The results of these tests are then sent to the GP to confirm the diagnosis and provide insights for appropriate management. Additionally, the service takes blood pressure measurements in patients at the request of their GP. Finally, the service represents an opportunity to promote and reinforce healthy behaviors [161,162,163].

Pharmacies that provide this service are entitled to a set-up fee of GBP 440 (EUR 522), a fee of GBP 15 (EUR 18) for each clinic blood pressure check and a fee of GBP 45 (EUR 53) for each appropriate provision of ABPM. Furthermore, pharmacies were eligible for incentive fees upon the provision of ABPM services over time (GBP 1000 (EUR 1186)) within their first year, followed by GBP 400 (EUR 474) in subsequent years if the specified thresholds are achieved [162,163].

#### 4.6.3. Portugal

According to Portuguese legislation, community pharmacies are allowed to provide diagnostic and therapeutic services, as well as POCT for HIV, HCV, and HBV [60]. To provide POCT for HIV, HCV, and HBV, pharmacists must be qualified to do so, and this service should encompass pre- and post-test counseling, as well as the referral systems to other healthcare institutions for positive cases [169].

A 2019 study found that pharmacists identified several key factors that facilitated the implementation of point-of-care testing. These included the speed of testing, confidentiality, the counseling offered to patients, initial pharmacist training, and the trust placed in pharmacists by the public. Conversely, the study also highlighted several barriers, such as the stigma associated with infections, the testing procedure itself, logistical challenges, and issues related to the referral process. Importantly, the study concluded that pharmacies serve as a crucial setting for screening initiatives [170].

Pharmacies also played an important role during the COVID-19 pandemic by offering rapid antigen testing for the virus. If the pharmacy complied with the relevant legislation, the cost of the test was reimbursed in full for a specified period. Otherwise, the pharmacy could charge up to EUR 10 per test [171].

A study also demonstrates that the involvement of pharmacies in the testing strategy improved accessibility, reduced the distance people needed to travel to access testing, increased the number of tests being conducted, and allowed significant health gains. Furthermore, pharmacies may offer analysis of biochemical and physiological parameters, including glucose, uric acid, blood pressure, body mass index, and others. This enables an assessment of the patient’s health, which can inform the subsequent pharmacological management of the patient [59]. The pricing of these services are determined by each pharmacy.

Despite the absence of protocolization with the SNS, community pharmacies represent an important setting for POCT. Indeed, over 90% of community pharmacies conduct regular POCT for a range of parameters, including blood pressure, cholesterol, body mass index, and glycaemia. Studies have demonstrated the relevance of pharmacy POCT in two key contexts: first, screening for individuals at high risk of fatal CVD and fatal cardiovascular events; and second, supporting ongoing disease management by improving blood pressure control [172].

Pharmacists play a pivotal role in POCT, as they are uniquely positioned to identify individuals who would benefit from being tested. In certain cases, pharmacists may refer patients to physicians for diagnosis and appropriate therapy. Additionally, they can provide patients with health advice, thus improving their health status.

### 4.7. Smoking Cessation

The WHO stated that “The tobacco epidemic is one of the biggest public health threats the world has ever faced, killing over 8 million people a year around the world”, including approximately 1.3 million non-smokers exposed to second-hand smoke. Tobacco use kills up to half of those who do not quit, and there is no safe level of exposure to tobacco smoke. The consumption of tobacco products is also associated with an increased risk of developing a range of diseases and disabilities, as well as causing damage to nearly every organ of the body as it causes cancer, heart disease, stroke, lung diseases, diabetes, COPD, certain eye diseases, among others [173].

It is estimated that approximately 80% of the world’s 1.3 billion tobacco users reside in low- and middle-income countries, where the prevalence of tobacco-related illness and mortality is the highest. Tobacco use contributes to poverty by diverting household spending to tobacco. This spending behavior is challenging to modify due to the addictive nature of tobacco [174,175].

Heated Tobacco Products (HTPs) are a relatively new category of tobacco products that generate aerosols containing nicotine and toxic chemicals by heating, rather than burning, a tobacco-containing device. While HTPs are often promoted as being less harmful than conventional cigarettes, there is currently no conclusive evidence to support this claim. Although certain toxic chemicals present in tobacco smoke are found at significantly lower levels in HTP aerosol, the aerosol also contains other harmful substances, some of which are present at lower levels or entirely absent in traditional cigarette smoke [174].

Among smokers who are aware of the dangers of tobacco, the majority express a desire to quit; consequently, counseling and medication can increase the likelihood of successfully quitting by a factor greater than two [174]. It is also important to note that quitting smoking is beneficial at any age [176].

Furthermore, tobacco use has a detrimental impact on the environment. The environmental impact of tobacco is significant throughout its entire lifecycle. This encompasses the cultivation and curing of tobacco, the manufacturing of tobacco products, their distribution, consumption, and the subsequent disposal of waste. Community pharmacists are well-positioned to provide effective smoking cessation treatment due to their accessibility to members of the community, by providing both advice on the appropriate utilization of smoking cessation products and behavioral support [177].

#### 4.7.1. USA

The CDC has identified cigarette smoking as the leading cause of preventable disease, disability, and death in the USA. There are several cessation support resources available, such as telephone-based resources (Quitlines), text messaging support, web-based support, smartphone apps, and cessation support programs. Pharmacists across all states are prepared to advise on the use of OTC tobacco cessation products and direct individuals to quitlines and local prescribers for additional support [178].

As of January 2024, 20 states have passed legislation authorizing pharmacists to prescribe nicotine replacement therapy (NRT) products or the seven FDA-approved cessation medications to adults. Tobacco cessation therapy can be prescribed in different ways, including population-based collaborative practice agreements, standing orders, state-wide protocols, or independent prescribing. In the latter two cases, pharmacists are not only permitted to prescribe but also have the ability to bill the patient’s insurance for any covered smoking cessation services in addition to the prescription medication dispensed [14,179].

The scope of the products covered under state-wide protocols differs across states. Some protocols include varenicline and bupropion, as well as non-prescription and prescription NRT products. To engage in autonomous prescribing of tobacco cessation products, pharmacists must meet minimum educational requirements [180].

As part of a study, a descriptive cross-sectional electronic survey was conducted among pharmacy personnel from a major American grocery store pharmacy chain. The results showed that 73 out of 79 respondents (92.4%) agreed that it would be beneficial for community pharmacists to offer tobacco cessation services. However, the most commonly cited barrier was a lack of time within the normal workflow, with 43 respondents (54.4%) identifying this as a significant challenge [181].

#### 4.7.2. England—Smoking Cessation Service (SCS)

The SCS is an advanced service designed to reduce morbidity and mortality associated with smoking, as well as to reduce health inequalities resulting from higher prevalence of smoking. The latter is achieved through patient’s referral from a hospital to a community pharmacy to continue the smoke cessation treatment, which includes appropriate medication and support, creating an additional capacity within the smoking cessation pathway [182,183,184].

In order to provide this service, pharmacies must notify NHS England of their intention in participating, have an adequate consultation room, have a carbon monoxide (CO) monitor, and have a standard operating procedure (SOP) in place. The service must be provided by a pharmacist or pharmacy technician, who have completed essential training and adhere to the SOP. The initial consultation should be conducted in person and a CO test should also be carried out. In addition, behavioral support and a supply of NRT (for a maximum of 2 weeks) is provided if deemed adequate, at no cost to the patient [183,184].

It should be noted that remote consultations are acceptable, as long as confidentiality is assured. Formal reviews are recommended at 4- and 12-weeks post-cessation. A successful cessation is defined as self-reported abstinence and a CO monitoring reading of <10 ppm at four weeks after cessation [183,184].

The patient’s GP should be notified of the service provided. Pharmacies involved in this service are reimbursed the cost of the NRT according to the Drug Tariff. In addition, they receive a set-up fee of GBP 1000 (EUR 1186), GBP 30 (EUR 36) for the first consultation, GBP 10 (EUR 12) for the interim, and GBP 40 (EUR 47) for the last [184].

#### 4.7.3. Portugal

The Portuguese legislation demands that health services, including community pharmacies, should disseminate information and health education on the adverse effects of tobacco consumption and the significance of both prevention and smoking cessation. This is achieved through the implementation of campaigns, programs, and initiatives [185].

Pharmacies may offer a shorter intervention, while patients with tobacco-related health issues should be accompanied by a team of specialized health professionals for a more intensive intervention. It is recommended that the smoking cessation service incorporates pharmacological, motivational, and behavioral components. A pharmacist may recommend OTC NRT as a first line of treatment, and, if unsuccessful, they may refer the patient to another health institution for an appropriate prescription and management [185,186,187].

Many community pharmacies in Portugal offer smoking cessation programs. These have shown good success rates, with approximately 20% of participants remaining abstinent after 12 months of the program, which consisted of pharmacological and behavioral interventions [188,189].

### 4.8. Pharmacists’ Prescription Authority

In some countries, pharmacists may be permitted to prescribe medications to enhance accessibility to healthcare and optimize treatment [190,191]. However, the specific models of pharmacist prescribing may vary across countries and between legislations [191].

#### 4.8.1. USA

In the U.S., pharmacists can prescribe medications through collaborative prescribing agreements that are either patient-specific or population-based, or through autonomous prescribing, which may follow statewide protocols or be unrestricted within defined therapeutic categories [14].

Collaborative prescribing:

- Patient-specific collaborative practice agreement: Between the patient, their provider(s), and the pharmacist. Typically used for chronic disease management for specific patients [192] (e.g., diabetes, hypertension, asthma [14]).
- Population-specific collaborative practice agreement: Between the provider(s) and the pharmacist, and services that may attend a broad patient populations. Typically used for acute care and chronic disease management [192] (e.g., treatment of influenza in patients that do not meet criteria for automatic referral [14]).

Autonomous prescribing:

- State-wide protocol: A qualified pharmacist may prescribe medications without direct supervision of a collaborating physician, and according to a published protocol by an empowered state body. Typically used in preventive care or for acute or self-limiting conditions that require no diagnosis or are easily diagnosed [192].
- Unrestricted category-specific authority: Pharmacists autonomously prescribe a medication without the supervision of a collaborating physician, for a legitimate medical purposes and within the pharmacist’s usual course of professional practice [192] (e.g., HIV PrEP and PEP, and emergency contraception in some states [193,194]).
- Autonomous and collaborative prescribing supplement each other, rather than being mutually exclusive [14].

#### 4.8.2. England

In the UK, pharmacists can prescribe medicines as independent or supplementary prescribers, provided they are registered as such [15,195].

- Independent prescribing: Pharmacists are authorized to assess patients without a prior diagnosis and determine the appropriate clinical management. This includes the ability to prescribe any medication considered clinically appropriate, with the exception of certain restricted substances [15,195].
- Supplementary prescribing: Pharmacists may prescribe medications typically reserved for physicians, in agreement with both the patient and the prescribing doctor, as part of a pre-established clinical management plan. This model supports continuity of care, allowing pharmacists to renew prescriptions and adjust the dosage or formulation to suit the patient’s needs [15,195].

##### Pharmacy First

The Pharmacy First service started in January 2024. It builds on the NHS Community Pharmacist Consultation Service, which started in October 2019. The objective of this service is to enable patients to be referred to a community pharmacy or to contact a pharmacy directly to receive care for a minor illness or for an urgent re-stock of their medication supply [196,197,198,199].

The Pharmacy First service encompasses three distinct categories of care: urgent medication supply, minor illness referral, and clinical pathways consultations. The first two categories are both subject to a referral requirement, while the latter can be conditional upon the suitability of the identified patients [196,197,198,199].

The Pharmacy First service allows community pharmacies to manage and complete episodes of care for seven common conditions in eligible patients, following predefined clinical pathways. These conditions include acute otitis media in patients aged 1 to 17 years, impetigo and infected insect bites in individuals aged 1 year and older, shingles in patients aged 18 years and older, and uncomplicated urinary tract infections (UTIs) in women aged 16 to 64 years. This service enables patients to access certain prescription medications directly from a pharmacy, without the need for an appointment with their general practitioner (GP) [196,197,198,199].

This service is expected to release GP’s appointments, enabling individuals to gain more timely and accessible care that meets the highest standards of safety and quality [196,197].

In order to implement this service, pharmacies must ensure the availability of consultation rooms that can be used for private and confidential appointments with patients. Furthermore, they must have adequate IT systems in place that allow for the Update Record functionality (enabling sharing of information about patient appointments outside of the general practice IT systems). Additionally, pharmacies must have the capacity to consult the patient’s health records (through GP Connect Access Record). Finally, pharmacy staff must possess the requisite level of expertise, including the ability to use an otoscope, familiarity with clinical pathways, protocols, and other relevant knowledge which may require additional training [196,197,198,200].

The Pharmacy First service is regarded as a pivotal initial step in acknowledging and adequately funding the vast quantity of healthcare advice that community pharmacies offer the public on a daily basis. Furthermore, this service aims to establish and fund community pharmacies as the first point of contact for healthcare advice [196,198].

The Pharmacy First Service can result in a number of outcomes, including the delivery of self-care advice to patients; supply of prescriptions or over the counter (OTC) medication (in line with the clinical pathway); referral of the patient to a separate commissioned pharmacy service within the pharmacy; or patient referral to a GP or other relevant health services (such as emergency services) [197].

Pharmacy contractors providing this service will be reimbursed according to arrangements set out in the Drug Tariff [197,198].

A fixed payment of GBP 2000 (EUR 2371) per pharmacy was initially made available prior to the launch of the service. This payment was to be withdrawn if the pharmacy did not reach a threshold of five clinical pathway consultations. A consultation fee of GBP 15 (EUR 18) is applied for each completed consultation. In addition to these fees, a fixed monthly payment of GBP 1000 (EUR 1186) is available to pharmacy owners who fulfill a minimum activity threshold in the number of clinical pathway consultations [198,199].

#### 4.8.3. Portugal

According to the legislation, in Portugal, only physicians (and dentists in certain cases) can prescribe medications [201]. Pharmacists may only recommend OTC and pharmacy-only medicines to treat minor illnesses [59,81,82,201,202].

##### Pharmaceutical Indication

A pharmaceutical indication is defined as the professional act undertaken by a pharmacist where a non-prescription medicine or health product is selected, and non-pharmacological measures are indicated with the aim of treating a minor health issue, following a clinical assessment by the pharmacist [59,81,82].

The pharmacist should conduct an interview with the patient in order to gain an understanding of their symptoms, their specific requirements, and their personal characteristics. In light of these considerations and with the pharmacist’s expertise in medicines, the pharmacist recommends the optimal course of action for the patient, which may include OTC medications, pharmacy-only medications (which must adhere to the INFARMED I.P. dispensing protocol), other health products, non-pharmacological counsel, or a referral to a different medical professional. This service should be registered, and it is not remunerated [59,81,82].

## 5. Discussion

Pharmacists represent a valuable resource for enhancing the efficacy of pharmacological interventions and facilitating health services, particularly during periods of crisis. They possess a comprehensive understanding of drug therapy, are widely regarded as a trusted source of information, are readily accessible to patients, and occupy a pivotal role within the medication use process. The delivery of pharmaceutical care also presents a crucial opportunity for pharmacists and is vital for ensuring the continued viability of the pharmaceutical profession [203,204].

The delivery of a wide range of healthcare services by pharmacies has the potential to enhance the health status of the population, improve the quality of life of patients, reduce the costs of treatments, and decrease the workload of physicians. Nevertheless, the services provided in pharmacies are not perceived as highly as they could be, and relatively few people make use of them [8].

Community pharmacies play a particularly important role in responding to the growing NCD pandemic by working within the primary healthcare network to provide early screening and testing, advanced counseling, and chronic disease management (including point-of-care measurements and medication management) [9].

Among the services explored in this work, Dispensing, Medication Review, Medication Reconciliation, and Medication Adherence services stand out. These services are likely to have the greatest impact on the management of NCDs. They prevent medication errors, have economic and quality-of-life benefits, optimize medication use, and improve health outcomes and disease management [15,110,125,133,134,135]. These services also have an impact on mortality by improving adherence and safety [205,206,207,208].

Services related to immunization and smoking cessation are of particular interest in the context of disease prevention and public health.

The involvement of community pharmacies in the administration of vaccines has been shown to have several positive outcomes, such as increased vaccination rates and positive economic and health effects [90,91,92,93,94].

Smoking cessation programs help patients to eliminate a major risk factor for the development of many NCDs [173]. Community pharmacists facilitate access to such programs and provide both advice on the appropriate use of smoking cessation products and behavioral support [177].

POCT services and programs are essential for the early detection and monitoring of NCDs. In this regard, pharmacists also play a role in the prevention of NCDs by providing health advice according to the POCT results [14,155].

Finally, hormonal contraceptive services have positive economic, social, and health impacts by improving access to contraceptives [140,141,142,143].

It is clear that, in the context of currently struggling healthcare systems, pharmacists are a valuable, pre-existing, skilled resource that could help support the healthcare system by providing a number of services leading to beneficial health and economic outcomes [5,6,7,9,209].

It seems reasonable to suggest that, in the future, pharmacy services and the role of pharmacists will assume a more prominent position in primary care. In alignment with the preceding argument, it seems probable that pharmacists will continue to provide patients with enhanced support in the management of their chronic conditions (e.g., providing more comprehensive MTM, being able to adjust the dosage of a patient’s prescription). Furthermore, it is likely that pharmacists will become more involved in disease prevention and wellness initiatives (e.g., conducting routine health checks and screenings). Additionally, pharmacists will potentially be able to treat more minor and acute illnesses and will likely be able to incorporate a stronger behavioral and mental health aspect into their practice (optimize medication therapy in collaboration with psychiatrists, administer depression screening questionnaires, and conduct behavioral therapy). Finally, they will be able to offer a greater number of services to elderly patients and collaborate with local social services organizations [210].

It is also important to highlight that progress in pharmacy services often relies on the development of more advanced and efficient IT systems tailored to identified needs [210], as well as on adequate and effective payment and reimbursement mechanisms to ensure the sustainability and expansion of services [211]. Additionally, patient awareness and collaboration with other components of the healthcare system are essential to the success of these advancements.

## 6. Conclusions

The three countries examined provide a range of similar cognitive, patient-centered pharmaceutical services, with a focus on prevention. However, notable differences exist in their healthcare systems, policies, and reimbursement of services across countries.

In England, the government’s policy of contracting out the provision of pharmacy services has the effect of standardizing the services offered, the quality standards, requirements and procedures, and improving accessibility and collaboration with state healthcare policies. England has a well-established system of reimbursement that recognizes and compensates community pharmacies for the services they provide [211]. Finally, one limitation of our analysis is that some of the information presented, while applicable to England, may not be relevant to other parts of the United Kingdom.

The legislative landscape in the USA is highly variable between states, which can result in discrepancies in the availability of services and the adherence to regulatory frameworks. This complexity poses challenges in the standardization of service provision, the quality of services offered, and the standards that govern them. The U.S. exhibits considerable heterogeneity in reimbursement models and services covered across different states [211].

In Portugal, community pharmacies offer a wide range of services, although mostly are not reimbursed. Portugal has a robust medication reimbursement system in place, although the vast majority of pharmacy services are subject to patient out-of-pocket expenses. Nevertheless, there has been an increase in government activity to meet the needs of the population, such as the reimbursement of medicines and some health products, as well as reimbursements to pharmacies for their Proximity Dispensing of Medication programs.

Several challenges regarding the implementation and expansion of pharmacy services remain. These include the need for training courses for pharmacists and pharmacy workers, licensing and pricing for the services, effective communication of service availability, access to patient information, and adequate reimbursement. In terms of the implementation of pharmacy services, England appears to have a more effective framework. This is characterized by the existence of established protocols and standard procedures, in addition to well-defined requirements and reimbursement structures, along with financial support for the implementation of these services and close cooperation with and support from the government.

Lastly it is anticipated that the role of community pharmacies will continue to evolve, becoming increasingly integrated into primary care and in healthcare systems. This will also involve countries learning from and adopting the successful practices of other countries. The cases of Portugal, England, and the USA show that community pharmacies can play a key role in improving healthcare, especially in managing chronic diseases and offering preventive services. For countries looking to implement similar services, it is important to establish clear legal frameworks, ensure pharmacists have access to patient health records, and promote collaboration with other healthcare professionals.

Moreover, a solid funding model is also crucial. England offers a strong example with its standardized, government-backed system, while Portugal shows how selective reimbursement can still support key services. The USA demonstrates the benefits of flexibility, but also the drawbacks of inconsistent regulation. Countries introducing these services should focus on proper training, IT integration, and raising public awareness to make them effective and sustainable.

## Figures and Tables

**Table 1:** Comprehensive overview of the existence of pharmaceutical services and the existence of reimbursement structures in each country.

Service	USA	England	Portugal
Dispensing Medication	✓ Medication Synchronization	✓ New Medicine Service ★ Repeat Prescription ★	✓ Proximity Dispensing of Medicines ★ Therapeutic Renewal
Immunizations	✓☆	✓☆	✓☆
Medication Review and Reconciliation	✓ Medication Therapy Management ★	✓ NHS Discharge Medicines Service ★	✓
Medication Adherence—Medication Packaging	✓	✓	✓ Individualized Medication Preparation (PIM)
Hormonal Contraception	✓/✗ (allowed in certain states) ☆	✓ NHS Pharmacy Contraception Service ★	✗
Point-Of-Care Testing	✓	✓ Lateral-Flow-Device Service ★ Hypertension Case-Finding Service ★	✓
Smoking Cessation	✓☆	✓ Smoking Cessation Service ★	✓
Pharmacists’ Prescriptive Authority	✓	✓ Pharmacy First ★	✗ Pharmaceutical Indication

✓—service exists. ✗—service does not exist. ★—there are reimbursement structures for the service. ☆—service might be reimbursed or not.

**Table 2:** Comprehensive overview of the effects and benefits of each pharmaceutical service.

Service	Effects/Benefits
Dispensing Medication	Opportunity for pharmacists to hold discussions with patients regarding their medications, thereby optimizing the utilization of medicines, enhancing health outcomes and the management and control of diseases
Immunizations	Increases immunization rates, convenience of vaccination, health access and equality, and results positive health effects
Medication Review and Reconciliation	Optimizes patient safety, effectiveness and efficiency of therapies, by identifying and resolving drug-related problems
Medication Adherence—Medication Packaging	Prevents medication errors, increases medication adherence
Hormonal Contraception	Increases prevention of unwanted pregnancies and empowers women
Point-of-Care Testing	Diagnoses certain diseases and involves patients in self-managing their heath conditions
Smoking Cessation	Increases accessibility to smoking cessation programs
Pharmacists’ Prescriptive Authority	Releases GP’s appointments, enables individuals to gain more timely and accessible care

## Data Availability

Data are contained within the article.

## Abbreviations

The following abbreviations are used in this manuscript:

ABMS	Appointment-Based Medication Synchronization
ABPM	Ambulatory Blood Pressure Monitoring
ACA	Affordable Care Act
AWP	Average Wholesale Price
BPMH	Best Possible Medication History
CLIA	Clinical Laboratory Improvement Amendment
CMS	Centers for Medicare & Medicaid Services
CO	Carbon Monoxide
CVD	Cardiovascular Disease
EC	Emergency Contraceptives
EHDS	European Health Data Space
FPL	Federal Poverty Level
GP	General Practitioner
HTP	Heated Tobacco Products
IT	Information Technology
LFD	Lateral Flow Device
MTM	Medication Therapy Management
MUR	Medication Use Review
NHS	National Health Service
NMS	New Medicine Service
NRT	Nicotine Replacement Therapy
OC	Oral Contraception
OTC	Over the Counter
PCS	Pharmacy Contraception Service
PGD	Patient Group Directions
PIM	Individualized Medication Preparation
POCT	Point-of-Care Testing
SCS	Smoking Cessation Service
SNS	Serviço Nacional de Saude
SOP	Standard Operating Procedure
UK	United Kingdom
U.S.	United States
USA	United States of America
WHO	World Health Organization
